# Lack of pronounced changes in the expression of fatty acid handling proteins in adipose tissue and plasma of morbidly obese humans

**DOI:** 10.1038/s41387-017-0013-x

**Published:** 2018-01-15

**Authors:** Ewa Anna Grzegorczyk, Ewa Harasim-Symbor, Bartlomiej Lukaszuk, Dorota Harasiuk, Barbara Choromanska, Piotr Mysliwiec, Malgorzata Zendzian-Piotrowska, Adrian Chabowski

**Affiliations:** 1 Department of Hygiene, Epidemiology and Ergonomics, Medical University of Bialystok, Bialystok, Poland; 2 Department of Physiology, Medical University of Bialystok, Mickiewicza 2C, 15-222 Bialystok, Poland; 3 Department of General and Endocrinological Surgery, Medical University of Bialystok, Bialystok, Poland

## Abstract

**Background/Objectives:**

Fatty acid handling proteins are involved in the process of accumulation of lipids in different fat tissue depots. Thus, the aim of the study was to estimate the expression of both fatty acid transport and binding proteins in the subcutaneous (SAT) and visceral adipose tissue (VAT) of patients with morbid obesity without metabolic syndrome, as well as the plasma concentrations of these transporters.

**Subjects/Methods:**

Protein (Western blotting) and mRNA (Real-time PCR) expression of selected fatty acid handling proteins was assessed in the visceral and subcutaneous adipose tissue of 30 patients with morbid obesity. The control group consisted of 10 lean age-matched patients. Plasma levels of fatty acid protein transporters were also evaluated using ELISA method. Moreover, total plasma fatty acid composition and concentration was determined by gas-liquid chromatography (GLC).

**Results:**

Significant increase in fatty acid translocase (FAT/CD36) mRNA (*P* = 0.03) and plasmalemmal (*P* = 0.01) expression was observed in VAT of patients with morbid obesity vs. lean subjects together with elevation of lipoprotein lipase (LPL), as well as peroxisome proliferator-activated receptor γ (PPARγ) in both examined compartments of adipose tissue. Moreover, in obese subjects plasma concentration of RBP4 was markedly elevated (*P* = 0.04) and sCD36 level presented a tendency for an increase (*P* = 0.08) with concomitant lack of changes in FABP4 concentration (*P* > 0.05).

**Conclusions:**

Fatty acid transport into adipocytes may be, at least in part, related to the increased expression of FAT/CD36 in the VAT of morbidly obese patients, which is accompanied by augmented expression of LPL, as well as PPARγ. Probably, alternations in plasma concentrations of RBP4 and sCD36 in obese patients are associated with “unhealthy” fat distribution.

## Introduction

Obesity is one of the most serious health problems in many countries and the prevalence of its occurrence is increasing worldwide^1^. Important pathophysiological basis of obesity is an increase in size and number of adipocytes due to the uptake and storage of the excess of energy in the form of lipids^2^. Subcutaneous obesity is referred to peripheral fat accumulation, whereas visceral obesity is generally related to abdominal fat accumulation within omental and mesenteric fat depots. Epidemiological studies have shown that visceral obesity compared to peripheral obesity is associated with a higher risk of obesity-related comorbidities such as insulin resistance, type 2 diabetes, cardiovascular disease, and dyslipidemia^3^.

Fatty acid (FA) transport into adipocytes apart from simple diffusion is a protein-mediated process. Protein transporters that were found to be highly involved in the facilitation of fatty acid uptake in adipose tissue are^4^ fatty acid translocase (FAT/CD36), plasmalemmal fatty acid binding protein (FABPpm), fatty acid transport protein (FATP-4), and cytosolic adipocyte fatty acid binding protein (FABP4). FAT/CD36 was identified as a key long-chain fatty acid (LCFA) transporter in fat tissue^5^. Furthermore, it was shown that FABPpm is expressed in a wide variety of tissues and its expression is up-regulated during preadipocyte differentiation. Other fatty acid transport proteins are also implicated in the FA accumulation in adipocytes. For instance, FATP-4 is known to affect triacylglycerols (TAGs) droplet size and other complex lipid pools formation. Once inside, fatty acids are bound by cytosolic fatty acid binding protein (FABPc), which stimulates not only FA absorption, but also its cytoplasmic redistribution^6^. FABP4 is the most abundant cytosolic isoform in adipocytes, which controls intracellular fatty acid transport and subsequent metabolism in fat tissue. The relative content of FABP4 in humans varies in different fat tissue depots and it has been shown that its mRNA level is related to the circulating insulin concentration in obese subjects^7^.

However, so far there are no data available on the expression of fatty acid handling proteins, which are involved in the accumulation of lipids in the visceral adipose tissue (VAT) and subcutaneous adipose tissue (SAT) of morbidly obese patients without metabolic syndrome. It is known that adipose tissue serves as a buffer for elevated consumption of dietary fatty acids. We suspect that the expression and/or content of LCFA protein transporters would be enhanced in different compartments (i.e., adipocytes and plasma) in order to compensate increased availability of lipids, and subsequently accumulate them as storage fraction (i.e., TAG) in adipocytes. Thus, the aim of our study was to assess the expression of membrane fatty acid handling proteins (FAT/CD36, FABPpm, FATP-4) at the mRNA and protein level in the SAT and VAT of patients with obesity, as well as the expression of lipoprotein lipase (LPL), peroxisome proliferator-activated receptor γ (PPARγ), cytosolic FABP4 and FABP5. Furthermore, the plasma concentrations of soluble CD36 (sCD36), FABP4 and retinol binding protein (RBP4) along with total plasma fatty acid composition have been determined.

## Materials and methods

The study included 30 obese patients (BMI > 40) (24 women and 6 men) without diabetes, hypertension, or other components of metabolic syndrome. General characteristic of patients are described in the Table 1. The individuals underwent bariatric surgery due to morbid obesity (BMI > 40). Control group consisted of 10 lean age-matched patients (BMI ≤ 26) (7 women and 3 men), who underwent elective laparoscopic cholecystectomy. All patients gave their informed consent to participate in the study. Moreover, all experiments were conducted in accordance with the guidelines of the Ethical Committee at the Medical University of Bialystok. Patients with acute inflammatory diseases and history of malignancy were excluded from the study. All the subjects were treated at the Department of General and Endocrinological Surgery of the University Hospital in Bialystok.

**Table 1:** General characteristic of obese and lean patients

Parameter	Lean patients	Obese patients	Reference values
Age	42,1 ± 10,5	46,3 ± 11,9	–
WHR	0,82 ± 0,1	0,93 ± 0,09	Male < 0,9; Female < 0,85
BMI (kg/m2)	24,5 ± 3,1	42,8 ± 5,9	18,5–24,99
CRP (mg/l)	5,7 ± 3,2	9,5 ± 5,3	(<10)
Glucose (mg/l)	98,2 ± 18,0	100,4 ± 18,6	(70–115)
ALT (UI/l)	26,9 ± 9,8	27,2 ± 10,1	(5–40)
AST (UI/l)	22,1 ± 6,0	21,0 ± 6,3	(5–40)
Cholesterol (mg/l)	190,2 ± 29,1	199,0 ± 35,1	(<200)
LDL (mg/l)	104,0 ± 20,2	128,6 ± 29,8	(<135)
TAG (mg/l)	120,2 ± 50,3	131,1 ± 60,4	(50–200)
HDL (mg/l)	50,1 ± 8,7	51,1 ± 9,9	(35–70)
WBC (103/µl)	6,2 ± 1,1	8,2 ± 1,6	(4,4–11,3)
RBC (106/µl)	4,6 ± 0,5	4,7 ± 0,4	(4,0–5,6)
HGB (g/dl)	14,0 ± 1,1	13,7 ± 1,2	(12–16)
HCT (%)	39,1 ± 3,2	41,0 ± 3,5	(34–47)
PLT (103/µl)	289,7 ± 40,3	303,8 ± 51,8	(140–440)
Fibrynogen (mg/dl)	315,2 ± 41,4	410,8 ± 58,0	(170–400)
INR	1,0 ± 0,06	0,9 ± 0,06	(0,9–1,3)

All parameters were determined using standard methods in the certified clinical laboratories of the University Hospital in Bialystok

Fasting blood samples were taken from control subjects and patients with obesity before surgery and were collected at EDTA-coated tubes. Next, the blood was centrifuged (10 min, 4000 r.p.m.) and the resultant samples were stored at −80 °C until further measurements. All parameters included in Table 1 were determined using routine methods in the certified clinical laboratories of the University Hospital in Bialystok (i.e., Department of Biochemical Diagnostics and Department of Hematological Diagnostics). At the end of the surgical intervention, the samples of SAT and VAT were taken from the upper part of the abdomen and promptly frozen in liquid nitrogen and stored at −80 °C.

### Real-time PCR

Total RNA was isolated from 30 mg of frozen tissue using NucleoSpin RNA (Macherey-Nagel) according to the producer’s instructions. First strand cDNA was generated using High Capacity cDNA Reverse Transkription Kit (Applied Biosystem). PCR products were obtained by amplification of cDNA using primers, as follows: forward: 5′-GGACGCTGAGGACAACAC-3′; reverse: 5′-GCCAGATTGAGAACTGTGAAG-3′ for FAT/CD36; forward: 5′-TAAGTTCAGCCGAGATGTC-3′; reverse: 5′-GTCATAATACCGATAACCTTGTAG-3′ for FABPpm; forward: 5′- GAAGGCAAAGGTGCGACAGT-3′; reverse: 5′- GCCGAACGGTAGAGGCAAA-3′ for FATP-4; forward: 5′-ATGTTCGTCATGGGTGTGAA-3′; reverse: 5′-GGTGCTAAGCAGTTGGTGGT-3′ for GAPDH. PCR was performed with SYBR Green JumpStart Taq ReadyMix (Sigma), using Bio-Rad Chromo4 system. Reaction mix in final volume of 25 µl consisting of 12,5 μl SYBR Green I, 3 µl cDNA, and 300 nM of each primer pairs. PCR was carried out under the following conditions: 15 s denaturation at 54 °C, 1 min annealing at 56 °C for FABPpm and GAPDH, 58 °C for FAT/CD36, 62 °C for FATP-4, 1 min extension at 72 °C for 40 cycles. PCR efficiency was examined by serial dilution of the template cDNA, and a melting curve was assessed after each reaction to verify PCR product specificity. A sample containing no cDNA was used as a negative control to verify the absence of primer dimmers. The results were normalized to GAPDH expression measured in each sample. Relative expression of genes was calculated according to Pfaffl method^8^.

### Isolation of plasma membrane fraction

Plasma membranes were isolated from adipose tissue according to Gargiulo et al.^9^. Briefly, samples of adipose tissue (250 mg) was placed in 2.5 ml of ice-cold SHB buffer (20 mM Tris, 1 M sucrose, 1 mM EDTA) containing protease and phosphatase inhibitor cocktail, as well as Triton-X100 (Sigma). The samples were homogenized in a Teflon-Glass homogenizer at 4 °C and then centrifuged (10 min, 500 g, 4 °C). Thereafter, the supernatant was layered on sucrose cushion (20 mM Tris, 1.12 M sucrose, 1 mM EDTA) and centrifuged (25 min, 101,000 g, 4 °C). Next, the pellet containing the plasma membrane fraction was suspended in SHB buffer and collected for Western blot analysis.

### Western blot analysis

Routine Western blotting procedures were used to detect protein content as described^10^. Briefly, the samples were homogenized in ice-cold RIPA (radioimmunoprecipitation assay) buffer containing phosphatase and protease inhibitors (Roche Diagnostics GmbH, Mannheim, Germany). Protein concentration was determined using bicinchonic acid method (BCA) with bovine serum albumin (BSA) as a standard. Next, homogenates were reconstituted in Laemmli buffer, separated by 10% sodium dodecyl sulfate-polyacrylamide gel electrophoresis, transferred onto nitrocellulose membranes and blocked (5% of nonfat dry milk or BSA). Then, the membranes were incubated overnight at 4 °C with the corresponding antibodies i.e., FAT/CD36 (1:1000, Abcam, UK), FATP-4 (1:500, Santa Cruz Biotechnology, USA), FABP4 (1:500, Santa Cruz Biotechnology, USA), FABP5 (1:500, Abcam, UK), FABPpm (1:1000, Abcam, UK), LPL (1:1000, Abcam, UK), PPARγ (1:500, Santa Cruz Biotechnology, USA), Na^+^/K^+^ pump (1:500, Santa Cruz Biotechnology, USA) and β-actin (1:500, Cell Signaling Technology, USA). Thereafter, the membranes were incubated with appropriate secondary antibodies conjugated with horseradish peroxidase (Santa Cruz Biotechnology, USA). Protein bands were visualized using an enhanced chemiluminescence substrate (Thermo Scientific, Rockford, IL, USA) and quantified densitometrically (ChemiDoc visualization system EQ, Biorad, Warsaw, Poland). Equal protein concentrations (50 µg) were loaded in each lane, which was confirmed by Ponceau S staining. Protein expression was normalized to β-actin. Finally, the control was set to 100% and the experimental groups were expressed relatively to the control.

### Plasma lipid composition

Measurement of total plasma fatty acid composition was performed according to method described in details by Glaser et al.^11^. Briefly, lipids in the presence of internal standard were extracted from plasma (250 µl) based on Folch method^12^ in chloroform/methanol (2:1, *v*/*v*) solution. Thereafter, methanolic HCl was added and samples were heated up to 85 °C for 45 min in order to synthetize methyl esters. After cooling, 1 ml of hexane was added, samples were centrifuged (900 g, 5 min), and upper phase was transferred to a new glass tube. Next, the extract was dried under nitrogen flow and lipids were dissolved in 50 µl of hexane. Individual fatty acid methyl esters were identified and quantified according to the retention times of standards by GLC (Hewlett-Packard 5890 Series II gas chromatograph, HP-INNOWax capillary column).

### ELISA analysis

Concentrations of sCD36, FABP4, and RBP4 were measured using commercially available kits for enzyme-linked immunoassay. All procedures were performed following the manufacturer’s instruction. sCD36 plasma concentration (ng/ml) was determined using ELISA kit provided by Aviscera Bioscience Inc. (USA). Eight fold dilution of each sample was required before the assay. Intra-assay and inter-assay coefficients of variations were less than 4–6% and less than 8–12%, respectively. Plasma concentration of the FABP4 (ng/ml) was measured using kit supplied by USCN Life Science Inc. (Wuhan, China). Intra-assay and inter-assay coefficients of variations were less than 10% and less than 12%, respectively. The RBP4 (µg/ml) ELISA kit was also provided by USCN Life Science Inc. (Wuhan, China). Intra-assay and inter-assay coefficients of variations were less than 10% and less than 12%, respectively. At the end of each measurement the intensity of colored product was measured in a hybrid multi-mode microplate reader (Synergy H1^TM^, BioTek Instruments, USA) at 450 nm.

### Statistical analysis

The results were statistically analyzed using Statistica 10 (StatSoft, Krakow, Poland). Data were analyzed using one-way analysis of variance (ANOVA) followed by post hoc Tukey’s test for groups with the normal distribution and variance homogeneity. Otherwise non-parametric Kruskal–Wallis with subsequent Wilcoxon’s pairwise test with Holm correction was applied. Statistical difference was indicated by a *P*-values < 0.05. Data were presented as mean or fold change ± SEM.

## Results

### Protein and mRNA expression of fatty acid transport proteins (FAT/CD36, FABPpm, and FATP-4)

**Fig. 1:**
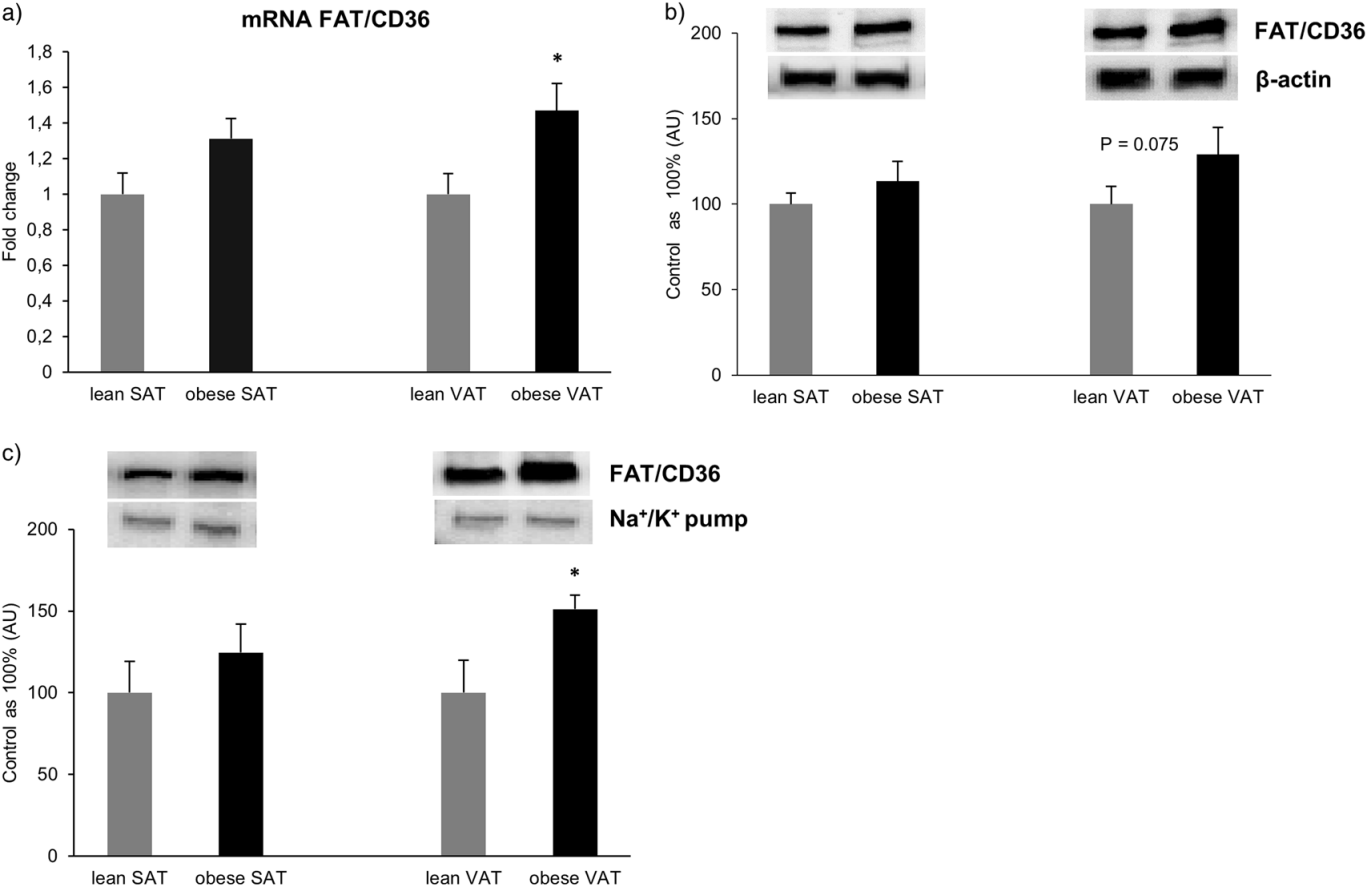
Expression of FAT/CD36 at the level of mRNA **a** as well as total protein (**b**) and plasma membranes (**c**) in subcutaneous and visceral adipose tissue of obese and control individuals. The data are expressed as fold change or percentage ± SEM. Control group was set as 100% (**b**) and (**c**). *P < 0.05 significant difference vs. respective control group (lean SAT or VAT)

In VAT, FAT/CD36 mRNA expression was significantly increased in patients with morbid obesity (+47%, *P* = 0.033; Fig. 1a) compared to the lean control. In SAT the expression of FAT/CD36 mRNA showed just a trend towards an increase in patients with obesity (+31%, *P* = 0.067; Fig. 1a). Moreover, we observed a tendency for an increase in the total protein expression of FAT/CD36 in VAT of patients with obesity (+23%, *P* = 0.075; Fig. 1b) compared to the lean patients’ VAT tissue. Additionally, FAT/CD36 plasmalemmal expression was also significantly elevated in obese VAT tissue (+51%, *P* = 0.019; Fig. 1c) in comparison with lean subjects.

**Fig. 2:**
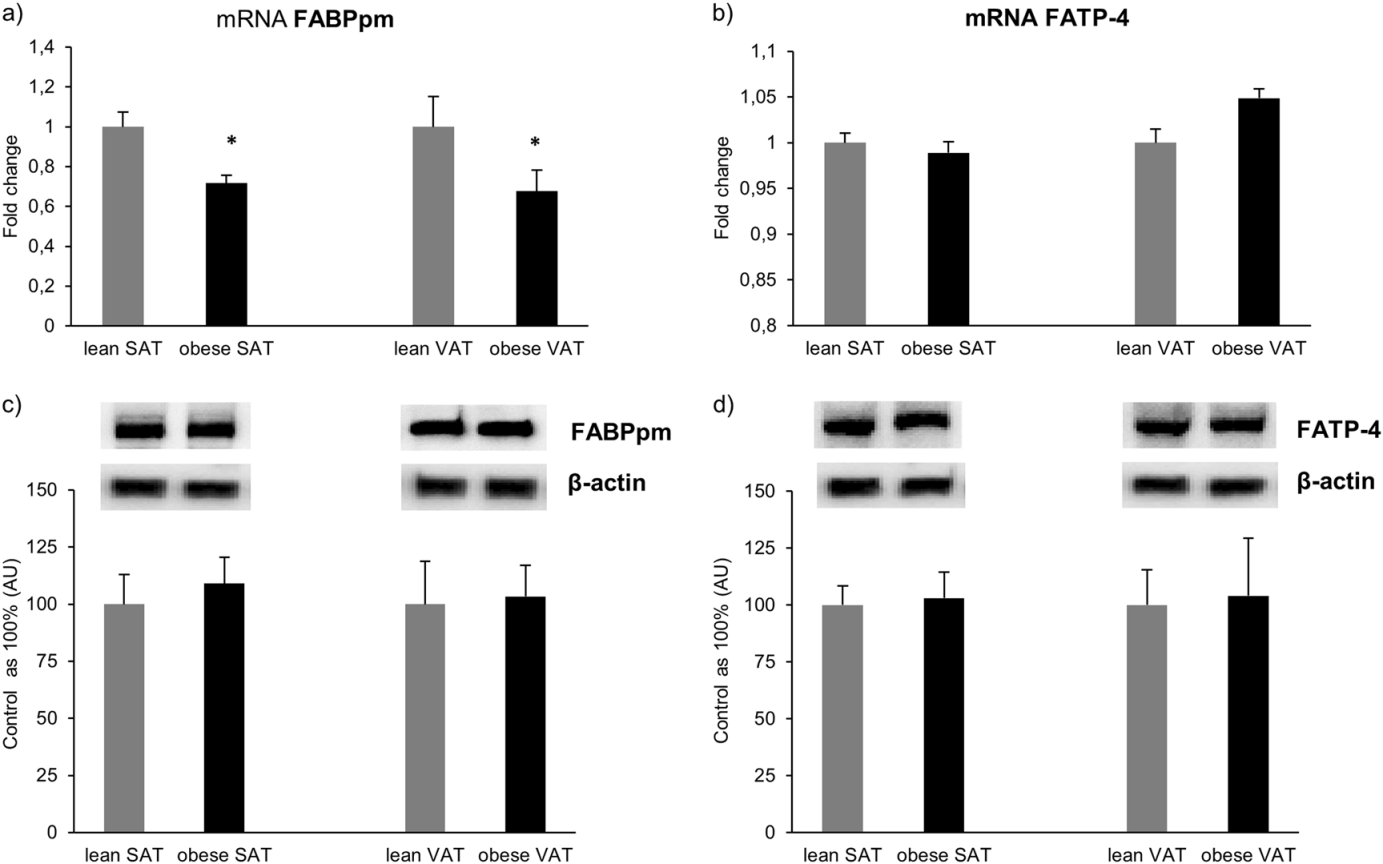
Expression of FABPpm and FATP-4 at the level of mRNA **a**, **b** as well as total protein (**c**), **d** in subcutaneous and visceral adipose tissue of obese and control individuals. The data are expressed as fold change or percentage ± SEM. Control group was set as 100% (**c**) and (**d**). *P < 0.05 significant difference vs. respective control group (lean SAT or VAT)

In contrast to FAT/CD36, we noticed a considerable decrease in the expression of FABPpm mRNA in obese SAT (−28%, *P* = 0.049; Fig. 2a), as well as obese VAT (−32%, *P* = 0.039; Fig. 2a) compared with the respective tissues in the control group. Nevertheless, there was no significant difference in the total expression of FABPpm at a protein level in both, the obese SAT and VAT (*P* = 0.1 and *P* = 0.13; Fig.2c) compared to the respective tissues in the control group.

In contrast to the above mentioned proteins, the expression of FATP-4 mRNA remained unchanged in both SAT and VAT of patients with obesity (*P* = 0.65 and *P* = 0.37; Fig. 2b) in comparison with the respective tissues in the control group. Thus, protein expression of FATP-4 in the tissues from patients with obesity was not altered compared to the lean patients’ SAT, as well as VAT (*P* = 0.14 and *P* = 0.1; Fig. 2d).

### Protein expression of cytosolic fatty acid binding proteins (FABP4 and FABP5), LPL and PPARγ

**Fig. 3:**
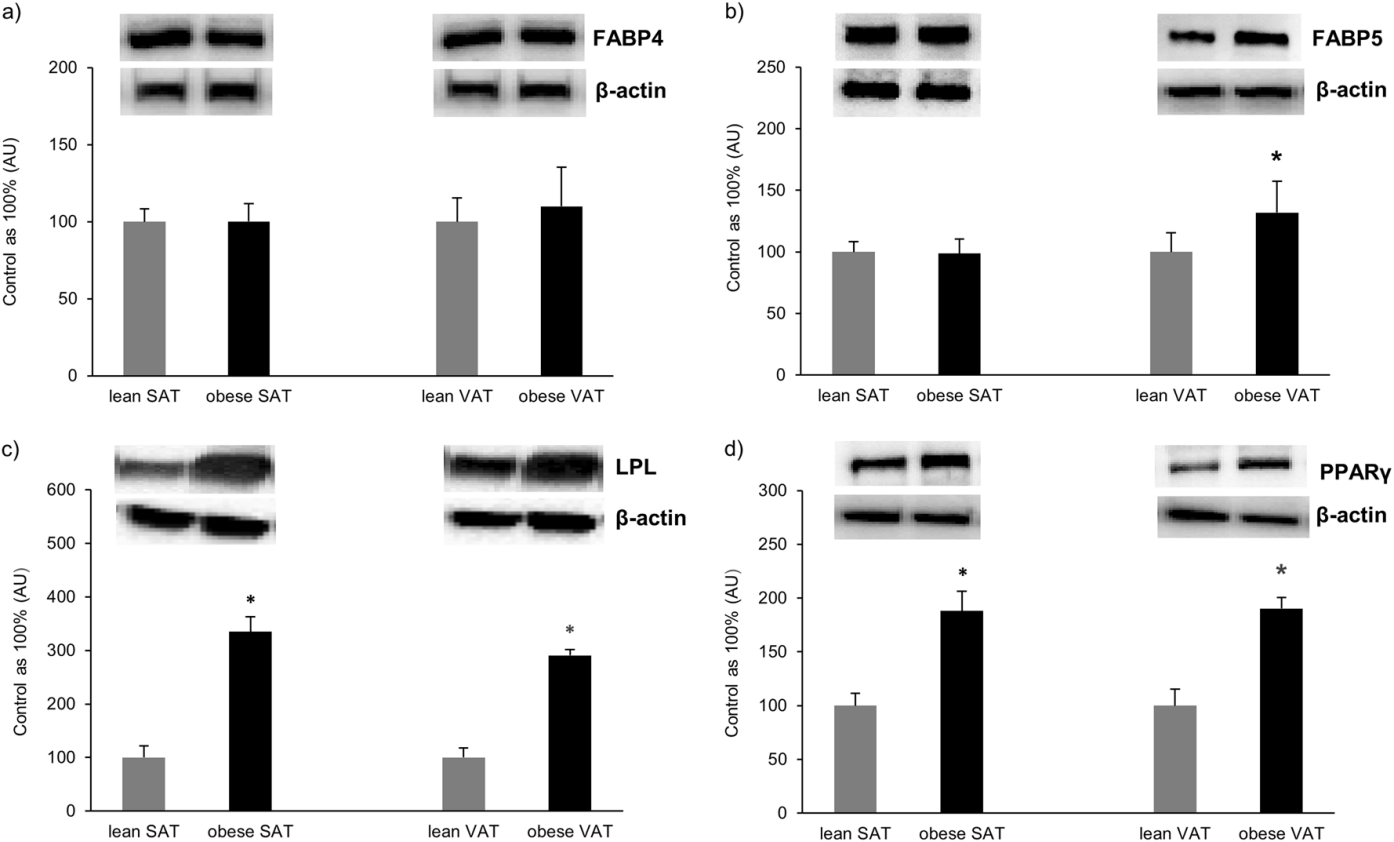
Total expression of cytosolic fatty acid binding proteins: FABP4 **a**, and FABP5 (**b**), along with LPL (**c**) and PPARγ (**d**) in subcutaneous and visceral adipose tissue of obese and control individuals. The data are expressed as percentage ± SEM. Control group was set as 100%. *P < 0.05 significant difference vs. respective control group (or obese VAT vs. obese SAT)

The expression of FABP4 remained unchanged in both SAT and VAT of the obese individuals compared with the corresponding tissues of the control group (*P* > 0.05; Fig. 3a). Nonetheless, the expression of FABP5 increased significantly in VAT of patients with morbid obesity compared to their SAT level (+36%, *P* < 0.05; Fig. 3b). Moreover, total expression of LPL, as well as PPARγ was markedly elevated in both SAT (+234%, *P* = 0.002; Fig. 3c and +88%, *P* = 0.001; Fig. 3d, respectively) and VAT (+191%, P = 0.005; Fig. 3c and +90%, *P* = 0.023; Fig. 3d, respectively) adipose tissue in obese individuals compared to control group.

### Plasma content of fatty acid binding proteins (FABP4, RBP4, and sCD36), as well as total fatty acid composition

**Fig. 4:**
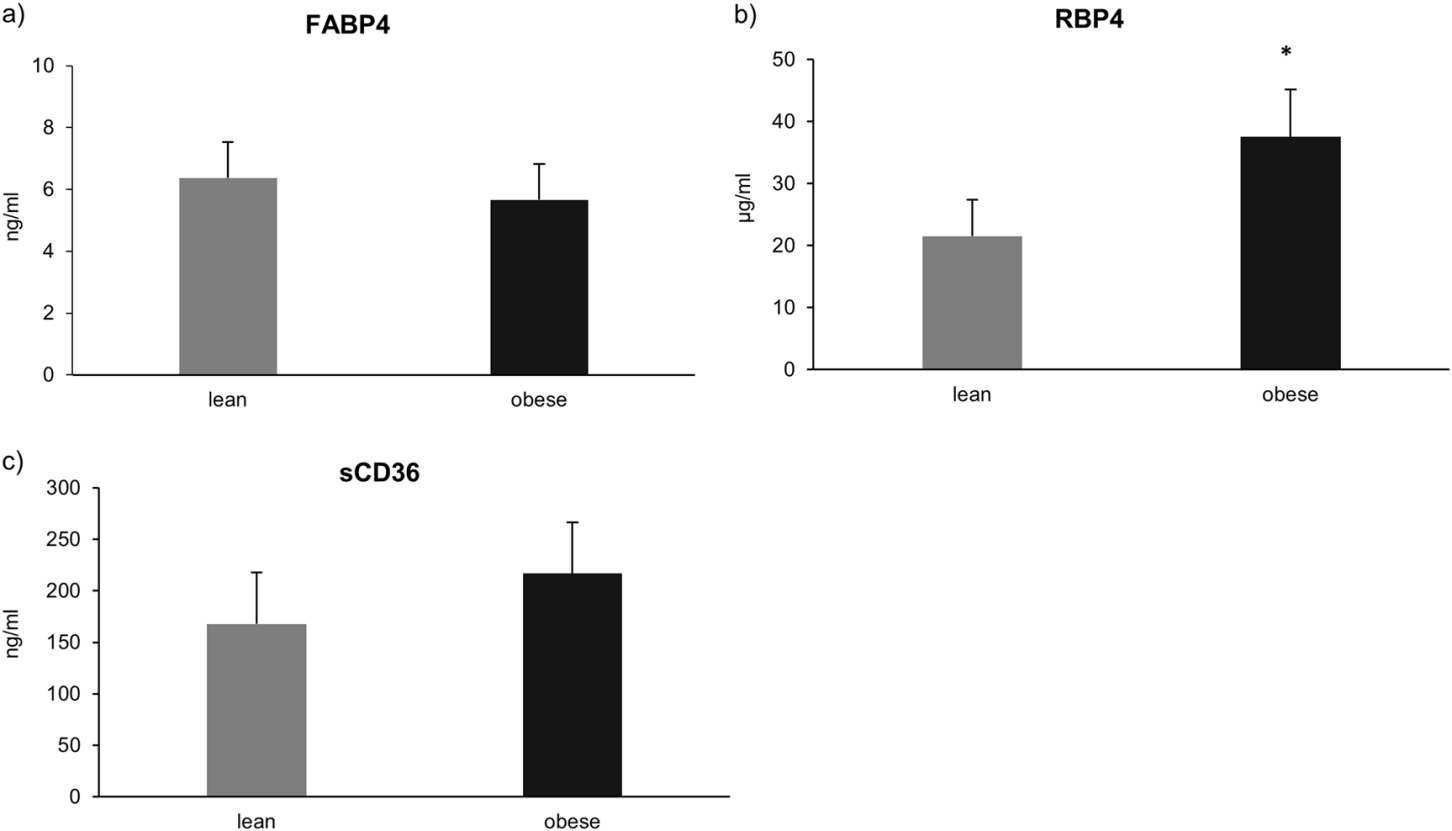
Plasma concentrations of FABP4 **a**, RBP4 (**b**) and sCD36 (**c**). The data are expressed as mean ± SEM. *P < 0.05 significant difference vs. respective control group

We did not observe any difference in the plasma concentration of cytosolic FABP4 in subjects with obesity compared to the lean ones (*P* = 0.09; Fig. 4a). Moreover, there was a trend towards an increase (+22%, *P* = 0.07; Fig. 4c) in the plasma concentration of sCD36 in the individuals with obesity compared to the lean controls, although the change did not reach significant level. Whereas, the plasma concentration of RBP4 of individuals with obesity was markedly elevated (+43%, *P* = 0.04; Fig. 4b) in comparison with the lean subjects. Interestingly, there was no difference in the total plasma fatty acid concentration, as well as in particular fatty acid species between obese patients and lean controls (*P* > 0.05; Table 2).

**Table 2:** Plasma total (from all lipid fractions) and individual fatty acids concentration (milligrams per liter) in obese and lean patients

Fatty acid	Lean patients	Obese patients
Myristic acid (C14:0)	25,02 ± 9,21	22,69 ± 9,32
Palmitic acid (C16:0)	687,58 ± 104,52	738,65 ± 162,37
Palmitoleic acid (C16:1)	68,65 ± 32,66	80,1 ± 30,37
Stearic acid (C18:0)	214,81 ± 25,52	205,03 ± 40,13
Oleic acid (C18:1)	629,13 ± 122,85	735,77 ± 188,69
Linoleic acisd (C18:2)	936 ± 180,19	789,18 ± 165,39
Arachidic acid (C20:0)	6,69 ± 0,59	6,65 ± 1,30
Linolenic acid (C18:3)	17,38 ± 5,06	16,56 ± 6,62
Behenic acid (C22:0)	13,23 ± 2,55	12,51 ± 3,66
Arachidonic acid (C20:4)	231,7 ± 60,07	246,21 ± 45,90
Lignoceric acid (C24:0)	15,58 ± 3,97	13,85 ± 3,73
Eicosapentaenoic acid (C20:5)	18,67 ± 6,71	16,88 ± 7,37
Nervonic acid (C24:1)	34,07 ± 9,92	36,71 ± 7,11
Decosahexaenoic acid (C22:6)	87,52 ± 20,85	93,43 ± 20,08
Total	2950,98 ± 434,19	3017,81 ± 510,34

The data are expressed as mean ± SEM

## Discussion

### Fatty acid membrane transport

In the previous studies, several authors have shown^5, 13–18^ that FAT/CD36 is a major regulator of LCFA transport across the plasma membrane in various metabolically active tissues, including adipose tissue. In our study, we demonstrated an increase in the expression of FAT/CD36 in obese patients (in VAT and to a lesser extend in SAT) compared to the lean controls. Similar data, although in rats, were reported by Luiken et al.^14^. These authors have presented FAT/CD36 mRNA expression, which was significantly increased in epididymal adipose tissue of obese Zucker rats. According to our study and others^14^, we can speculate that up-regulation of FAT/CD36 gene expression may be responsible at least partially for the excessive lipid accumulation in adipose tissue. Interestingly, observed augmented expression of FAT/CD36 on different levels (mRNA and plasma membranes) in VAT compared to SAT in patients with morbid obesity may also confirm that VAT is more metabolically active tissue than SAT in this group of people. However, Bower et al.^19^ compared the mRNA and protein expression of FAT/CD36 in the VAT of obese Afro-American women (AAW) and Caucasian women (CAW). These authors revealed that the mRNA expression of FAT/CD36 in AAW tends to be higher compared to CAW in the VAT, which was also followed by increased uptake of LCFA in this group. Although we examined only morbidly obese Caucasian race subjects in our study, we could also observe the elevation in FAT/CD36 plasmalemmal and mRNA expression in VAT compared with the lean group. On this bases, it is likely that the VAT of the subjects with obesity might have had a greater potential for the uptake of LCFA, similarly to other authors^19^. Furthermore, we indicate increased expression of PPARγ together with LPL in both examined depots of adipose tissue in obese individuals. It is likely, that in our study PPARγ, a major regulator of adipogenesis, is partially responsible for enhanced expression of FAT/CD36 and LPL, since it serves as a transcription factor for these genes in mature adipocytes^20^. Interestingly, we did not observe alternations in the total plasma content of fatty acids from different lipid fractions (it represents plasma fatty acids status) in morbidly obese patients (Table 2). Probably, it resulted from significantly increased LPL along with FAT/CD36 (especially plasmalemmal) expression in both examined compartments of adipose tissue, which compensated fatty acids dietary intake in a long-term basis by enhancing TAGs accumulation in adipose tissue. Studies conducted on mice with increased expression of LPL and high-fat diet induced obesity^21^ revealed protective role of this enzyme in glucose and insulin tolerance. It is in line with present study, since our patients in spite of morbid obesity were insulin sensitive and did not display symptoms of metabolic syndrome. Moreover, in the current study FABPpm mRNA expression was substantially lower in the SAT, as well as VAT in the individuals with obesity compared to the lean subjects. Similar data were noticed by Lappas et al.^22^, in which the authors have presented that FABPpm mRNA expression in the SAT of obese (normal glucose tolerant pregnant women) has also reached significant decrease. Probably, in our case the decrease in mRNA was not followed by a decline in FABPpm expression at the protein level. Furthermore, in our study there was no change in FATP-4 mRNA and protein expression in the SAT and VAT in subjects with obesity compared to the respective tissues of the lean control. Thus, we can speculate that this protein may not be essential for fatty acid uptake in adipocytes. This notion found confirmation in a mouse model of adipocyte Fatp4 gene inactivation^23^, which revealed that lack of FATP-4 expression did not affect fatty acid transport into the cells. Taken together, our and others^5^ observations indicate that FAT/CD36 is a major LCFA protein transporter, which is responsible for augmented fatty acid uptake especially in VAT depot and subsequent accumulation of TAGs, as energy storage.

### Cytosolic FABPs

FABPc stimulate not only FA desorption from biological membranes but also their cytoplasmic transport to the destination place^6^. Thus, FABPc can be defined as not only the acceptors but also as a transport proteins^24^. In our study, there was no change in FABP4 protein expression in SAT and VAT in patients with morbid obesity compared to the lean controls. Animal studies have shown that FABP4 deficiency improved insulin resistance, lipid metabolism, atherosclerosis, and inflammatory state, under conditions of genetic or dietary obesity^25, 26^. Thus, it is not surprising that we did not observe any alternations in FABP4 expression in different depots of adipose tissue, since examined patients with morbid obesity were sensitive to insulin. Lack of changes in FABP4 (plasma concentration and tissue expression) and increased PPARγ expression in morbidly obese subjects is in agreement with studies revealing negative feedback between expression of FABP4 and PPARγ content in adipocytes^27^. The other form of adipocyte FABPs is known as a keratinocyte lipid-binding protein or mal1 (FABP5), which is also released by macrophages and found in the skin, lung, brain, testis, and lens^28–31^. Based on our study, we may only hypothesize that increased expression of FABP5 in obese VAT compared with obese SAT could be due to the fact that VAT is more metabolically active than SAT in obesity. Furthermore, we cannot exclude that observed rise is gender-dependent, since the majority of examined obese subjects were women. However, additional studies are needed to explain the exact role of FABP5 in human obesity.

### Plasma FABPs

In the current study, we noticed lack of changes in FABP4 plasma concentration in obese patients compared with the lean subjects. This is consistent with the results of total FABP4 expression in VAT, as well SAT tissues, since we did not noticed any alternations in morbidly obese individuals. Moreover, other authors^32^ have presented close correlation between plasma levels of FABP4 and its expression in adipose tissue in different fat depots, suggesting that adipose tissue is a major contributor of circulating FABP4. Furthermore, it was shown that plasma level of FABP4 can be an indicator of metabolic syndrome, type 2 diabetes mellitus, and increased lipolytic activity of adipose tissue. e.g., weight loss^33, 34^. Our results are in agreement with the above mentioned, since patients examined in our study did not exhibit any components of metabolic syndrome in spite of morbid obesity. Therefore, our findings confirmed the hypothesis that plasma FABP4 level can serve as a biomarker of insulin resistance or metabolic syndrome.

Moreover, we revealed an upward trend for elevated plasma concentration of sCD36 in patients with obesity compared to the lean controls. Recently, it has been shown that, circulating sCD36 level was positively associated with the size of abdominal fat depots in individuals with morbid obesity^35^. Additionally, it was found that the level of sCD36 is associated not only with the ‘unhealthy fat distribution’ but also with the circulating levels of TAG. Moreover, previous studies have presented that the plasma sCD36 content is significantly higher among obese and diabetic subjects^36, 37^. Additionally, Knöstgard et al.^35^ has demonstrated that adults with morbid obesity who underwent bariatric surgery presented significant weight loss and improvements in metabolic disturbances, along with a concomitant reduction in circulating sCD36. The results of our study and others^35^ are consistent with other findings and support an important role of sCD36 in the development of complications associated with diet-induced obesity^38, 39^.

Retinol binding protein (RBP4) is another peptide secreted from adipocyte and liver. In our study, we have demonstrated that RBP4 concentration was significantly elevated in the plasma of patients with obesity compared with non-obese group, which was consistent with previous investigation^40^. Similarly to our results, it was shown that increased level of RBP4 positively correlates with body mass index, as well as waist circumference^41, 42^. There are contradictory data concerning close connection between raised values of circulating RBP4 and development of insulin resistance. However, it was indicated that increased level of RBP4 changes glucose metabolism and therefore may lead to insulin resistance^43^. Researchers have also demonstrated^40, 44^ that circulating RBP4 level was linked to the concentration of plasma triacylglycerols and cholesterol in such states as obesity, type 2 diabetes, and cardiovascular implications. Thus, it means that RBP4 affects not only glucose pathway but also lipid metabolism. Probably, in our case elevated plasma levels of RBP4, as well as sCD36 (a trend) are indicators of obesity along with developing insulin resistance state and cardiovascular disorders.

For the first time we have shown the status of different fatty acid handling proteins (in plasma and adipose tissue) that are involved in adipocyte’s lipid metabolism in morbidly obese patients without metabolic syndrome symptoms. Our findings indicate on FAT/CD36, as a major protein transporter enhancing FA uptake in adipocytes, especially in VAT. Moreover, in the present study we showed a relevant role of FAT/CD36, LPL, and PPARγ in buffering excessive fatty acid dietary intake in morbidly obese but healthy individuals. Undoubtedly, the role of FABP4, sCD36, and RBP4 as the potential biomarkers of obesity, metabolic syndrome, and cardiovascular diseases was also confirmed in our studies.
